# Various Subtypes of EGFR Mutations in Patients With NSCLC Define Genetic, Immunologic Diversity and Possess Different Prognostic Biomarkers

**DOI:** 10.3389/fimmu.2022.811601

**Published:** 2022-02-21

**Authors:** Youming Lei, Kun Wang, Yinqiang Liu, Xuming Wang, Xudong Xiang, Xiangu Ning, Wanbao Ding, Jin Duan, Dingbiao Li, Wei Zhao, Yi Li, Fujun Zhang, Xiaoyu Luo, Yunfei Shi, Ying Wang, Depei Huang, Yuezong Bai, Hushan Zhang

**Affiliations:** ^1^ Department of Geriatric Thoracic Surgery, The First Affiliated Hospital of Kunming Medical University, Kunming, China; ^2^ Department of Thoracic Surgery, Anning First Peoples Hospital affiliate to Kunming University of Science and Technology (Kunming Forth People’s Hospital), Kunming, China; ^3^ Department of Pulmonary and Critical Care Medicine, The First Affiliated Hospital of Kunming Medical University, Kunming, China; ^4^ Department of Thoracic Surgery, The Third Affiliated Hospital of Kunming Medical University, Kunming, China; ^5^ Department of Thoracic Surgery, The First Peoples Hospital of Yunnan Province, Kunming, China; ^6^ Department of Oncology, Yan’an Hospital Affiliated to Kunming Medical University, Kunming, China; ^7^ Department of Thoracic Surgery, Yan'an Hospital Affiliated to Kunming Medical University, Kunming, China; ^8^ Department of Oncology, Yunnan Provincial Hospital of Traditional Chinese Medicine, Kunming, China; ^9^ The Medical Department, 3D Medicines Inc., Shanghai, China

**Keywords:** NSCLC, EGFR - epidermal growth factor receptor, immune microenviroment, prognosis, biomarkers

## Abstract

Based on data analysis of 9649 Chinese primary NSCLC patients, we calculated the exact proportion of EGFR subtypes in NSCLC and evaluated the TMB level, PD-L1 expression level and tumor immune microenvironment among different EGFR mutation subtypes. Postoperative follow-up data for 98 patients were collected and analyzed. The results showed that several uncommon EGFR mutation subtypes have a higher proportion of TMB-high or strong positive PD-L1 expression than the total EGFR mutation group. In addition, different subtypes have different characteristics related to the immune microenvironment, such as G719 mutations being associated with more CD8^+^ T cell infiltration into tumors; except for EGFR 19del, CD8^+^ T cell infiltration into tumors of other EGFR mutation subtypes were similar to that of wildtype EGFR. Moreover, follow-up results revealed that components of the immune microenvironment have prognostic value for NSCLC patients, with different prognostic biomarkers for NSCLC patients with and without EGFR mutations. These results suggest that patients with different EGFR mutations need to be treated differently. The prognosis of NSCLC patients may be assessed through components of tumor immune microenvironment, and ICIs treatment may be considered for those with some uncommon EGFR mutation subtypes.

## Introduction

Lung cancer is a complex disease consisting of a variety of molecular and histological types of clinical relevance. The incidence of lung cancer is rising globally. GLOBOCAN estimated 2.09 million new cases in 2018, which is higher than the 1.8 million new cases reported in 2012. The 5-year survival of lung cancer was reported in 2019 to be 19.4% (1). Clinical diagnosis of lung cancer is divided according to pathology into small cell lung cancer (SCLC) and non-small-cell lung cancer (NSCLC). NSCLC can be further classified into squamous cell carcinoma (SCC), adenocarcinoma (ADC), large-cell lung carcinoma (LCLC), and other types, including salivary gland-type tumors and sarcomatoid carcinomas, etc. (2). EGFR mutations are detected in 15% of lung adenocarcinomas in the United States, and the most common mutations occur in exon 19 and exon 21 (3, 4); in China, however, the proportion of EGFR mutations in lung cancer is very different from that in other regions. In this study, we used data for 9649 Chinese primary NSCLC patients to calculate the exact ratio of different EGFR mutation subtypes reported to be associated with the efficacy of tyrosine kinase inhibitors (TKIs) and immune checkpoint inhibitors (ICIs). Large-scale molecular profiling helps to identify potential molecular targets that can be applied in treating patients with specific lung cancers and facilitates a more refined molecular classification of lung cancer.

Programmed cell death protein 1/programmed cell death ligand 1 (PD-1/PD-L1) act to suppress activation of T lymphocytes, and anti-PD-1/PD-L1 therapy has gained great success in the treatment of several solid tumors, such as lung cancer. In addition, biomarkers that can be used to predict response to immunotherapy, to optimize patient benefit and to minimize negative effects have been widely explored and utilized, including PD-L1, TMB, MSI/dMMR (5, 6), and components of the tumor immune microenvironment. PD-1/PD-L1 immunotherapy strategies have recently been explored for NSCLC treatment. Before examining the potential of immunotherapy for each EGFR mutation subtypes of NSCLC, we attempted to unveil the genetic and immunologic landscape of NSCLC harboring different EGFR mutations.

Heterogeneity is an important feature of tumors, and in addition to cancer cells, a wide range of immune cells can infiltrate into human tumor tissues, including both innate and adaptive immune cells. Immune cells within the tumor microenvironment, termed the tumor immune microenvironment (TIME), play important roles in tumor evolution. It is now increasingly accepted that cancer cells interact closely with the components of the TIME, and in turn, the characteristics of the TIME can affect tumor response to immunotherapy. Moreover, the prognostic significance of components in the TIME, such as CD8^+^ T cells, has been revealed for several cancers (7, 8). Among adaptive immune cells, tumor-infiltrating lymphocytes (TILs) have been explored widely (9). By definition, TILs include several immune cells, such as T-cells, B-cells, and NK cells (10). The components and characteristics of a specific tumor microenvironment can be considered predictive biomarkers and, to some extent, provide clues regarding the potential application of ICIs.

## Material and Methods

### Clinical Specimens

The Formalin-Fixed Paraffin-Embedded (FFPE) tissues samples from 9649 primary NSCLC patients who have underwent next-generation sequencing (NGS) in a laboratory accredited by the Clinical Laboratory Improvement Amendment (CLIA) and College of American Pathologists (CAP) (3D Medicines Inc., Shanghai, China) from June 2015 to October 2020 were analyzed. Formalin- Hematoxylin and eosin (H&E) staining was used to evaluated tumor cell content of FFPE tissue sections using. Only samples with a tumor content of 20% and above were allowed for subsequent analyses. The written informed consent was obtained from all included patients. The postoperative follow-up data of 98 NSCLC patients were collected, the median follow-up is 27.3 months., as of now, three of these patients have died. Characteristics of these patients have been listed in the table (**Table 1**).

**Table 1:** Patient characteristics in the follow-up cohort.

Characteristics	N	%
**Total**	98	
**Gender**		
Male	53	54.08
Female	45	45.92
**Age**		
>60	42	42.86
<60	56	57.15
**Histology**		
Squamous-cell carcinoma	5	5.10
Adenocarcinoma	89	90.82
Adeno-squamous carcinoma	2	2.04
Large cell carcinoma	2	2.04
**Stage**		
1	46	46.94
2	30	30.61
3	22	22.45
**EGFR mutation**		
Without (EGFR-)	41	41.84
With (EGFR+)	57	58.16
**Subtypes of EGFR mutation**		
EGFR exon 19 deletion (19del)	17	17.35
EGFR L858R	14	14.29
G719A\C\S	12	12.24
S768I	13	13.27
Others (N468K, E709A, N771delinsGY, H870R, P772_H773dup, I740_K745dup, V774M, A767_V769dup, R451H, D761Y and amplification)	15	15.31
At least two EGFR mutation subtypes	14	14.29
**Available TIME analysis**	39	39.80
**Available TMB analysis**	32	32.65
**Postoperative therapy**		
Targeted therapy	13	13.27
Chemotherapy	34	34.69

### Tissue Processing and Genomic DNA Extraction

FFPE tissue sections were placed in a 1.5 microcentrifuge tube and deparaffinized with mineral oil. Then incubated the samples with proteinase and lysis buffer K overnight at 56° C t until the tissue was fully digested. Subsequently, the lysate was incubated at 80°C for 4 hours to reverse formaldehyde crosslinks. Then followed the manufacturer’s protocol, isolated genomic DNA from tissue samples using the ReliaPrep™ FFPE gDNA Miniprep System (Promega) and quantified using the Qubit™ dsDNA HS Assay Kit (Thermo Fisher Scientific).

### Library Preparation and Targeted Capture (for WES)

Samples of 70 patients were underwent whole exome sequencing (WES). An S220 focused ultrasonicator (Covaris) was used to spear DNA extracts (30-200 ng) to 250 bp fragments using a. Libraries were subsequently prepared using the KAPA Hyper Prep Kit (KAPA Biosystems) according to the manufacturer’s introduction. The fragment size distribution and concentration of each library were determined using a LabChip GX Touch HT Analyzer (PerkinElmer) and a Qubit 3.0 fluorometer (Thermo Fisher Scientific), respectively. Briefly, 500 ng of indexed DNA libraries were pooled to obtain 2 μg of DNA, which was then mixed with Human Cot-1 DNA and xGen Universal Blockers-TS Mix and dried down in a SpeedVac system. The Hybridization Master Mix was added, followed by incubation at 95°C for 10 min. Four microliters of the exGen Exome Research Panel v1.0 (IDT) were then added and the mixture was incubated at 65°C overnight. Target regions were captured following the manufacturer’s instructions. The size distribution and concentration of the final library were determined using a LabChip GX Touch HT Analyzer (PerkinElmer) and a Qubit 3.0 fluorometer (Thermo Fisher Scientific), respectively.

### Library Preparation and Targeted Capture (for Tissue-Based Targeted Panel Sequencing)

An S220 focused ultrasonicator (Covaris) was used to spear DNA extracts (30-200 ng) to 250 bp fragments using an. Then KAPA Hyper Prep Kit (KAPA Biosystems) was used to prepare libraries were prepared. The fragment size distribution and concentration of each library were determined using a LabChip GX Touch HT Analyzer (PerkinElmer) a Qubit 3.0 fluorometer (Thermo Fisher Scientific), respectively. For targeted capture, indexed libraries were subjected to probe-based hybridization with a customized NGS panel targeting whole exons of 733 cancer-related genes (**Supplementary Table 1**), where the probe baits were individually synthesized 5′ biotinylated 120 bp DNA oligonucleotides (IDT). According to the annotation of UCSC Genome RepeatMasker, repetitive elements were filtered out from intronic baits. The xGen^®^ Hybridization and Wash Kit (IDT) was used for hybridization enrichment. Briefly, 500 ng indexed DNA libraries were pooled to obtain a total amount of 2 μg of DNA. The pooled DNA sample was then mixed with human cot DNA and xGen Universal Blockers-TS Mix and dried down in a SpeedVac system. The Hybridization Master Mix was added to the samples and incubated in a thermal cycler at 95°C for 10 min, before being mixed and incubated with 4 μl of probes at 65°C overnight. The target regions were captured following the manufacturer’s instructions. The size distribution and concentration of the final library were determined using a LabChip GX Touch HT Analyzer (PerkinElmer) and a Qubit 3.0 fluorometer (Thermo Fisher Scientific), respectively.

### DNA Sequencing, Data Processing, and Variant Calling (for Tissue-Based Testing)

The captured libraries were loaded onto a NovaSeq 6000 platform (Illumina) for 100 bp paired-end sequencing with a mean sequencing depth of 35000X. Using the Burrows-Wheeler Aligner (v0.7.12) to map the raw data of paired samples (an FFPE sample and its normal tissue control) to the reference human genome hg19. Deleted PCR duplicate reads and used Picard (v1.130) and SAMtools (v1.1.19) to collect sequence metrics, respectively. Using an in-house developed R package to detect Somatic single nucleotide variants (SNVs) to execute a variant detection model based on binomial test. Local realignment was performed to detect indels. Filtered variants though their unique supporting read depth, base quality and strand bias, as described before (11). Then filtered all variants using an automated false positive filtering pipeline to ensure specificity and sensitivity at an allele frequency (AF) of ≥ 5%. Indels and single-nucleotide polymorphism (SNPs) were annotated by ANNOVAR against the following databases: 1000Genome, dbSNP (v138) and ESP6500 (population frequency > 0.015). Only stopgain, missense, frameshift and non-frameshift indel mutations were retained. Gene rearrangements and copy number variations (CNVs) were detected as described earlier (11).

### PD-L1 Expression by Immunohistochemistry (IHC) 22C3 Antibody

FFPE tissue sections were subjected to assessment of PD-L1 expression using the PDL1 IHC 22C3 pharmDx assay (Agilent Technologies). In brief, FFPE tissue sections were dried, and fixed on positively charged slides at 56 to 60°C for 1 hour. With antigen repaired, then placed the slides in the Autostainer Link 48 platform (Dako) to incubate with the anti-human PD-L1 monoclonal antibody, clone 22C3 pharmDx, then incubated with the secondary antibody, and subsequent a substrate-chromogen solution (DAB). Tumor Proportion Score (TPS), as described previously (12, 13), was calculated to evaluate PD-L1 expression level.,

### Tumor Microenvironment (TME) by Multiplex Immunofluorescence (mIF)

Multiplex immunofluorescence staining was conducted using the PANO 7-plex IHC kit (Panovue). Primary antibodies targeting CD8 (clone C8/144B), CD56 (clone 123C3), HLA-DR (clone EPR3692), CD68 (clone BP6036) and PanCK (Cocktail) were sequentially applied to FFPE tissue slides, subsequently, incubated with horseradish peroxidase-conjugated secondary antibody and tyramide signal amplification. The slides were heat-treated in a microwave after each round of tyramide signal amplification. Cell nuclei acids were stained with 4′-6′-diamidino-2-phenylindole (DAPI, SIGMA-ALDRICH) once all immune cells had been labelled. A Mantra workstation, capture fluorescent spectra at 20 nm wavelength intervals from 420 nm to 720 nm with an absolute magnification of ×200 and ×100 and fixed exposure time, was used to scan multiplex stained slides. Then superimposed all scans for each slide to obtain a single image.

Images of monoplex stained and unstained slides were applied to subtract the spectrum of each tissue autofluorescence and fluorophore, respectively. The inForm Image Analysis Software (PerkinElmer) was applied to create a spectral library required for multispectral unmixing. This spectral library was used to re-constructure slide images without autofluorescence. The quantity of immune cells including macrophages, CD8^+^ T cells, and NK cells were calculated as the number of positively stained cells per square millimeter and percentage of positively stained cells in all nucleated cells.

### TMB

For data processing and reads mapping, please refer to “DNA sequencing, data processing, and variant calling (for tissue-based testing)”. TMB was defined as the count of synonymous and nonsynonymous somatic SNVs and indels in detected coding regions, excluding driver mutations. All indels and SNVs in the coding region were considered, including missense, silent, stop gain, stop loss, in-frame and frameshift mutations.

### MSS/MSI

100 microsatellite loci were selected for determination of MSI and for each assay, the top 30 loci with the best coverage were included for evaluation of final MSI score. An in-house developed R script was applied to assess the distribution of read counts among different repeat length of each microsatellite locus. A MSI score was defined as the percentage of unstable loci. The sample of MSI-H was the one with MSI score ≥ 0.4, otherwise it was MSS.

### Statistical Analysis

Data were analyzed using the GraphPad Prism software (version 7.01). Data were presented as the mean ± standard error of the mean (SEM). Differences between two groups were analyzed using the student unpaired t test or an unpaired t test with Welch’s correction. analysis of variance was used to investigate more than two groups. Univariate Cox proportional hazards models of survival and biological baseline variables were built to estimate hazard ratios (HRs) with a 95% CI. Survival curve was assessed using Kaplan-Meier and log-rank test, and p-value of less than 0.05 (two- tailed) was considered as significant difference.

## Results

### Frequencies of Different Subtypes of EGFR Mutations in Chinese Primary NSCLC

**Figure 1:**
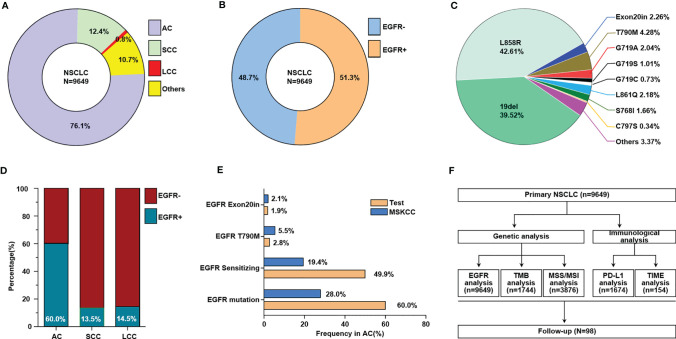
The frequencies of different subtypes of EGFR mutations in Chinese primary NSCLC. **(A)** The proportion of different histologic types of NSCLC, others including adenosquamous carcinoma, sarcomatoid carcinoma, typical carcinoid, atypical carcinoid, lyphoepithelionma-like carcinoma, etc. **(B)** The ratio of NSCLC patients with EGFR mutation and without EGFR mutation. **(C)** The ratio of different EGFR mutation subtypes in NSCLC patients. **(D)** The ratio of patients with EGFR mutation and without EGFR mutation in different histologic types of NSCLC. **(E)** Comparison of selected gene alteration frequencies in this research and MSKCC cohort. **(F)** Flow charts for data analysis in this research. AC, adenocarcinoma; SCC, squamous cell carcinoma; LCC, large cell carcinoma.

Different histologic types of NSCLC in China were calculated based on data for 9649 primary NSCLC patients (**Figure 1A**). EGFR mutations were detected in 51.3% of Chinese NSCLC patients, as illustrated in the pie chart in **Figure 1B**. Different subtypes of EGFR mutations in primary NSCLC patients were evaluated (**Figure 1C**), and more EGFR mutations were found in adenocarcinoma than in squamous cell carcinoma and large cell carcinoma (**Figure 1D**), and. Furthermore, we compared the frequency of several of EGFR mutations in our research to that in the prospective MSKCC cohort (14). (**Figure 1E**). A total of 9649 samples of NSCLC patients were used to analyze EGFR mutations, and then some of them were subjected to subsequent genetic and immunological analysis (**Figure 1F**).

### Some EGFR Mutation Subtypes Displayed Higher TMB Levels, With a Higher Proportion of TMB-High Patients

**Figure 2:**
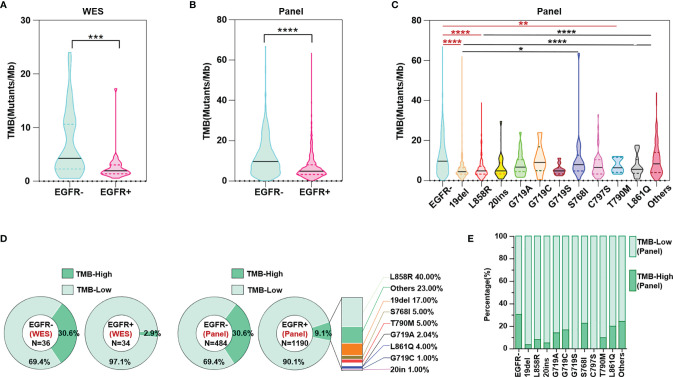
TMB level evaluated both through WES and panel sequencing. **(A)** WES calculated TMB level in NSCLC patients with and without EGFR mutation. **(B)** Panel sequencing calculated TMB level in NSCLC patients with and without EGFR mutation. **(C)** Panel sequencing calculated TMB level in NSCLC patients of different EGFR mutation subtypes. **(D)** Ratio of TMB high or low evaluated by WES and panel sequencing. **(E)** Ratio of TMB high or low evaluated by panel sequencing in different subtypes of EGFR mutation. *means p < 0.05, **means p < 0.01, ***means p < 0.001, ****means p < 0.0001.

Both the results of WES and panel sequencing that covered the whole exome of 733 cancer-related genes showed lower TMB levels in NSCLC patients with than in those without EGFR mutations (**Figures 2A, B**). Based on WES, TMB≥10 mutations/Mb was defined as TMB-High, and otherwise TMB-Low. The proportion of TMB-Low detected by WES in those without EGFR mutation was 69.4%. We hypothesize that if the panel is sensitive enough to detect the TMB, then the proportion of this part detected by this panel should be similar to the WES detection result; that is, the proportion of TMB-Low detected by the panel in group without EGFR mutation should be 69.4%. The same ratio of TMB-Low was set as the EGFR wildtype group as counted by WES, and the cutoff of TMB-High and TMB-Low by the panel was 14.5 mutations/Mb. Based on these results, subsequent analysis showed that TMB levels differed among EGFR mutation subtypes. NSCLC patients without EGFR mutation showed higher TMB-levels than in 19del, L858R and T790M groups. However, no significant difference between the group without EGFR mutation and the other EGFR mutation group, except for 19del, L858R and T790M, was found. Moreover, the 768I and “Others” groups showed a higher TMB than the 19del or L858R group. In summary, the results indicated that NSCLC patients with uncommon mutations have TMB levels similar to those of EGFR wildtype patients (**Figure 2C**). The results of both WES and panel sequencing revealed lower proportion of TMB-High in NSCLC patients with EGFR mutation. The descending order in TMB-High proportion was L858R, other uncommon mutations, 19del, S768I, T790M, G718A, LL861Q, G719C and 20ins (**Figure 2D**). In the group of other uncommon mutations, S768I, L861Q and G719A mutation groups, TMB-High was observed in more than 15% of patients (**Figure 2E**).

### The Level of Tumor PD-L1 Expression Was Different Among EGFR Mutation Subtypes, and Differences in Immune Cell Infiltration Were Associated With Different Levels of PD-L1 Expression

**Figure 3:**
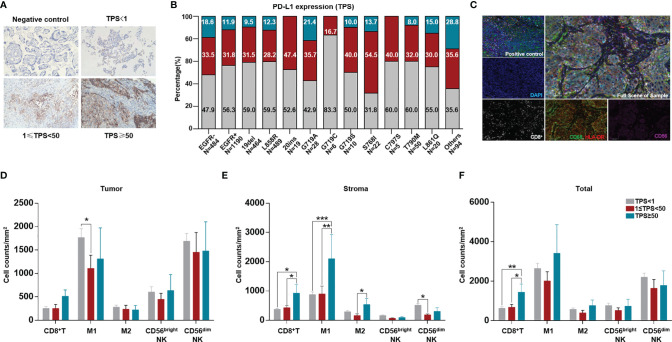
Comparation of tumor PD-L1 expression and ratio among different EGFR mutation subtypes and difference of TIME among different TPS groups. PD-L1 expression was quantified as negative (TPS<1%), intermediate positive (1%≤TPS<49%), and strong positive (TPS≥50%) for available cases and tabulated across different EGFR mutation subtypes. **(A)** Representative staining of PD-L1 expression through IHC (200X). **(B)** Comparation of PD-L1 expression between tumors with and without EGFR mutation, and among different EGFR mutation subtypes. **(C)** Ratio of patients with MSI-H in Chinese NSCLC. A total of 3876 were available for count the ratio of MSI-H. A total of 141 NSCLC patients were available for analysis of both PD-L1 expression and TIME. **(D)** Representative staining for CD8^+^T cells, CD56^bright^ and CD56^dim^ NK cells, CD68^+^HLA-DR^+^ M1, CD68^+^HLA-DR^-^ M2 TAM. CD8 (white), CD56 (purple), CD68 (green), HLA-DR (red), panCK/S100(cyan), DAPI (blue). **(E, F)** different immune cell counts in intra-tumoral region, stroma, and sum of both. *means p < 0.05, **means p < 0.01, ***means p < 0.001.

PD-L1 expression was detected in 1674 tumor samples *via* IHC. Among available tumor samples, a higher proportion of tumors without EGFR mutations had positive PD-L1 expression (TPS≥1) and strong positive PD-L1 expression (TPS≥50). Tumors without EGFR mutations, with EGFR G719S, S768I and other uncommon EGFR mutations, had a higher proportion of positive PD-L1 expression than the left subtypes (**Figures 3A, B**).

Furthermore, we evaluated the TIME among different TPS groups. Although no difference in CD8^+^ T cells infiltrating into tumors was found in different TPS groups, in the stroma and in tumor+stroma, more CD8^+^ T cells were found in the group with strong PD-L1 expression (TPS≥50). More M1 macrophages infiltrated into the tumor in the PD-L1-negative group than in the PD-L1-positive group; in the stroma, more M1 macrophages were found in the group with strong PD-L1 expression. No differences in other immune cells were found in the tumor, stroma, or tumor+stroma (**Figures 3C–F**).

### Differences in Infiltration of Various Immune Cells Were Found Among Tumors With Different EGFR Mutation Subtypes

**Figure 4:**
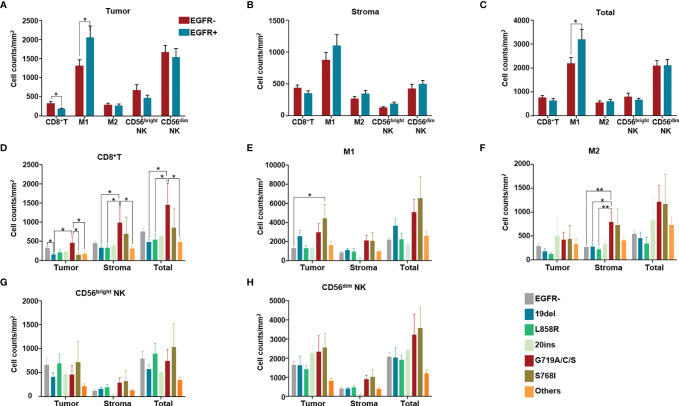
Immune cell counts both in tumor (intra-tumoral region) and stroma of 154 NSCLC patients. Immune cell counts both in tumor (intra-tumoral region) and stroma of 154 NSCLC patients of EGFR wild type and with different EGFR mutation subgroups. **(A)** Numbers of different immune cells in tumor were compared between without and with EGFR mutation groups. **(B)** Numbers of different immune cells in stroma were compared between without and with EGFR mutation groups. **(C)** Numbers of different immune cells in total (stroma and tumor) were compared between without and with EGFR mutation groups. **(D–H)** Numbers of CD8^+^ T cells, M1, M2 TAM, CD56bright NK cells, CD56dim NK cells in tumor, stroma, in total (tumor and stroma) were compared among patients of different EGFR mutation subgroups. (in the group termed as “other uncommon mutations”, contained EGFR amplification, EGFR p.E709A, EGFR p.E709K, EGFR p.E709V, EGFR p.F254L, EGFR p.H773dup, EGFR p.H870R, EGFR p.I740_K745dup, EGFR p.L747P, EGFR p.N468K, EGFR p.R451H, EGFR p.R776C, EGFR p.R776L and EGFR p.S768_D770dup). *means p < 0.05, **means p < 0.01.

We evaluated the difference in immune cell infiltration between tumors with and without EGFR mutations. Among CD8^+^ T cells, M1 (CD68^+^HLA-DR^+^) tumor-associated macrophages (TAMs), M2 TAMs (CD68^+^HLA-DR^-^), CD56^bright^ NK cells and CD56^dim^ NK cells, less CD8^+^ T cell infiltration was found in the EGFR mutation group than in the group without EGFR mutation (p<0.05). In addition, more M1 TAMs infiltrated in the tumor and in tumor+stroma were found in the EGFR mutation group than in the EGFR wildtype group (p<0.05) (**Figures 4A–C**).

The main differences among CD8^+^ T cells, M1 TMAs, M2 TAMs, CD56^bright^ NK cells and CD56^dim^ NK cells were regarding counts of CD8^+^ T cells and TAMs for different EGFR mutation subtypes (**Figures 4D–H**). In detail, compared with the group without EGFR mutations, only the 19del group showed fewer infiltrated CD8^+^ T cells (p<0.05) (**Figure 4D**). Comparing different EGFR mutation subtypes with the EGFR wildtype group, no significant differences were found in the numbers of CD8^+^ T cells in either the stroma or in tumor+stroma. However, differences in the numbers of CD8^+^ T cells in the tumor, stroma, and tumor+stroma were found for some EGFR mutation subtypes; for example, more CD8^+^ T cell infiltration in the tumor was found in the EGFR G719A/C/S mutation group than in the 19del and S768I groups (**Figure 4D**). Except for more M1 infiltration in the S768I mutation group, there were no differences in the number of M1 macrophages in the other EGFR mutation subtype groups compared with the EGFR wildtype group (**Figure 4E**). Despite no significant differences in M2 infiltration, more M2 infiltration was present in stroma in the EGFR G719A/C/S group than in the EGFR wildtype (p<0.05) and 19del (p<0.05), L858R mutation groups (p<0.01) (**Figure 4F**).

### Different Prognostic Biomarkers Were Found for Patients With and Without EGFR Mutations

**Figure 5:**
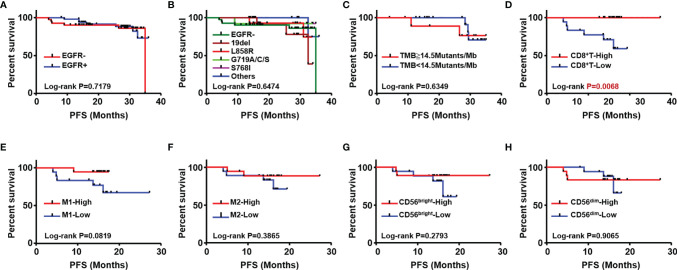
Analysis of association with patients’ outcome. Kaplan Meier estimates for progression-free survival (PFS); patients were stratified according to with (EGFR+) and without EGFR mutation (EGFR-) **(A)** different subtypes of EGFR mutation **(B)** TMB **(C)** CD8+T **(D)** M1 **(E)** M2 macrophages **(F)**, CD56bright NK **(G)** and CD56dim NK cells **(H)**.

**Figure 6:**
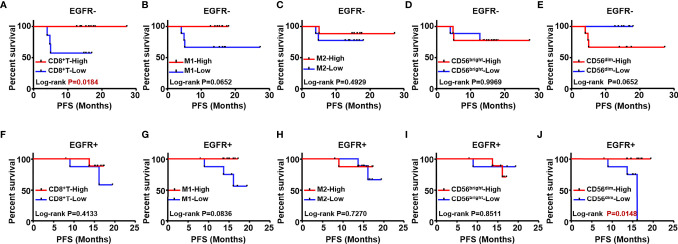
The prognostic value of different immune cells was evaluated in NSCLC patients with and without EGFR mutation. Kaplan Meier estimates for PFS; Patients without EGFR mutation (EGFR-) were stratified according to CD8+T **(A)**, M1 macrophages **(B)** M2 **(C)** CD56bright NK **(D)** and CD56dim NK **(E)** Patients with EGFR mutation (EGFR+) were stratified according to CD8+T **(F)** M1 macrophages **(G)** M2 **(H)** CD56bright NK cells **(I) **and CD56dim NK cells **(J)**.

To explore prognostic biomarkers for NSCLC, we followed up 98 postoperative NSCLC patients. These patients were stratified according to without and with EGFR mutation (EGFR-/+), TMB level, immune cells, including CD8^+^ T cells, M1/M2 macrophages, CD56^bright^ and CD56^dim^ NK cells. Log-rank progression-free survival (PFS) analysis was performed by using cutoff values of the top ½ density of tumor-infiltrating immune cells, such as CD8^+^ T cells (CD8^+^ T-high defined as patients with the top ½ CD8^+^ T cell density in the tumor; others defined as CD8^+^ T-low), M1/M2 macrophages and CD56^bright^, CD56^dim^ NK cells, in NSCLC tissues (**Figures 5A–H**). According to the results, EGFR mutation (**Figures 5A, B**), TMB level (**Figure 5C**), and components of the TIME (**Figures 5D–H**), CD8^+^ T cells were associated with different prognoses; in detail, a longer PFS was associated with CD8^+^ T cell-high (p<0.01) (**Figure 5D**). We further analyzed the potential prognostic significance of the TIME in NSCLC patients with and without EGFR mutations, and identified different prognostic biomarkers (**Figures 6A–J**). The prognostic significance of CD8^+^ T cells was only observed for patients without EGFR mutations (p<0.05) (**Figure 6A**); for patients with EGFR mutations, a longer PFS was found for the CD56^dim^ NK cell-high cohort than the CD56^dim^ NK cell-low cohort (p<0.05) (**Figure 6J**). Thus, NSCLC patients with and without EGFR mutations have different prognostic biomarkers.

## Discussion

Although EGFR is reported to be a major driver oncogene in lung cancer in Asia, especially adenocarcinoma, the exact proportion of Chinese NSCLC patients with EGFR mutations varies by report, and the difference may be related to the limited number of patients previously evaluated (3, 15). We recalculated the precise proportion of EGFR mutation and different EGFR mutation subtypes based on data for 9649 Chinese primary NSCLC patients. We compared the frequency of several of EGFR mutation types in lung adenocarcinoma in our research, including total EGFR mutations, EGFR sensitizing mutations, T790M mutation and exon 20 insertion, to that in the prospective MSKCC cohort (14). This result confirmed that the mutation frequency of the Chinese population is significantly different from that of populations in other regions previously reported. This suggests that for the Chinese population, clinical trials, treatments and clinical management strategies that are different from other populations should be given in the future practice.

ICIs have poor activity in NSCLC with driver alterations, and they have been excluded from the NCCN Guide for System Therapy of NSCLC. However, in ATLANTIC, a phase 2, open-label, single-arm trial, the effect of durvalumab treatment was assessed in patients with NSCLC; cohort 1 comprised EGFR+/ALK+ NSCLC patients, and the results for this cohort showed that EGFR^+^ NSCLC patients with ≥25% of tumor cells expressing PD-L1 benefit from ICIs (16). Responses to ICIs in NSCLC patients with EGFR 19del, L858R, T790 M and EGFR ex20ins have also been evaluated, including patients with the common EGFR exon del 19 or L858R mutation who exhibited ORRs to ICI <20% and PFS less than 3.5 months (17). However, another study showed that compared 212 lung cancers without EGFR mutation, outcomes of ICI treatment were worse in patients with EGFR19del but similar in those with EGFR L858R; in contrast, EGFRT790M status did not affect response to ICI treatment (18). The results of a cohort of 58 patients treated with PD-1/PD-L1 inhibitors revealed that NSCLCs harboring EGFR mutations are associated with poor response, and such mutations were speculated to be associated with low rates of concurrent PD-L1 expression and CD8^+^ T cells within the tumor microenvironment (19). Furthermore, patients with EGFR exon 20 insertions (20ins) benefit less from ICIs than those without 20ins (20). Compared with NSCLC patients without targetable oncogenes, patients with EGFR 20ins NSCLC have better outcomes with platinum chemotherapy but derive less benefit from ICIs, as explained by lower levels of TMB and PD-L1 expression (20). These studies may indicate that not all patients with EGFR mutations show a lack of response to ICI treatment. Overall, comprehensive analysis of PD-L1 expression, TMB and even CD8^+^ T cell infiltration would help to better evaluate response to ICI therapy. Indeed, we should distinguish the genetic, immunologic and even clinical heterogeneity of each EGFR mutation subtype, as different subtypes may have different responses to ICIs. MSI-H is another important biomarker approved for ICI treatment in solid tumors (21), and we analyzed MSI-H in Chinese patients with NSCLC. Although more patients with MSI-H were found in the group with than in the group without EGFR mutations, similar with previous report (21, 22), only a very small portion of NSCLC cases were MSI-H (<1%) (**Supplementary Figure 1**). Therefore, other factors and mechanisms should be considered, and we should identify the specific characteristics of each subtype and analyze more TIME factors. We assessed both genomic and immunophenotypic characteristics of almost all EGFR subtypes that are reported to be related to different responses to TKIs and ICIs therapy, and based on our results, different strategies should be recommended for NSCLC patients with different EGFR mutation subtypes.

PD-L1 expression and MSI-H/dMMR have been recognized by the FDA as predictive biomarkers of immunotherapy response, furthermore, based on efficacy data from KEYNOTE-158 (NCT02628067) (23), the FDA granted accelerated approval to pembrolizumab for the treatment of patients with solid tumors of TMB-High (TMB≥10 Mutants/Mb) (23). In addition, a meta-analysis revealed TMB≥10 mutations/Mb to be significantly associated with enhanced objective response rates for immunotherapy (24). Therefore, we also defined WES-detected TMB≥10 mutations/Mb as TMB-high and WES-detected TMB<10 mutations/Mb as TMB-low. Following the principle that the proportion of TMB-Low in the Chinese population detected and calculated by WES and the panel should be consistent, we calculated that our 733-gene panel TMB cutoff equivalent to the WES TMB cutoff of 10 mutations/Mb should be 14.5 mutations/Mb (**Figure 2D**). In the ensuing analysis, we used 14.5 mutations/Mb as the TMB cutoff of our 733-gene panel; that is, we considered TMB≥14.5 mutations/Mb as TMB-High and others as TMB-Low. Although previous evidence has shown that EGFR 19del and L858R are common mutations in NSCLC patients carrying EGFR mutations, our results indicates that 19del and L858R account for 39.52% and 42.61% of NSCLC cases with EGFR mutations, respectively (**Figure 1D**). ICIs have no/limited activity in EGFR^+^ NSCLC, and almost all clinical trials exclude EGFR^+^/ALK^+^ patients, which may be because previous trials mainly enrolled these common mutations (accounting for at least 80% of EGFR mutations), which have lower TMB levels. As our results demonstrated, at least some cases of uncommon EGFR mutations, such as S768I and G719, should be considered for ICIs treatment because the TMB levels with these mutations were similar to those of the EGFR wildtype group.

The poor response to ICIs was previously partially explained as a lower TMB or PD-L1 expression level (25). Nevertheless, some recent evidence suggests that the predictive power of PD-L1 or TMB with regard to response to ICIs is limited in NSCLC patients with EGFR common mutations (26).Chen et al. proposed a classification system of human cancer based on both PD-L1 expression and TIME to search for potential situations suitable for immunotherapy (27). Therefore, identifying the type of TIME is necessary for guiding immunotherapy. We detected infiltration of CD8^+^ T cells, NK cells, and M1 and M2 TAMs, as based on some recent evidence. For example, changes in immune subpopulations between pretherapy and on-therapy samples from 68 patients with advanced melanoma revealed numerous changes in the immune response. In detail, an increase in the number of CD8^+^ T and NK cells and a decrease in M1 macrophages were associated with response to therapy (28). The density of T-cells infiltrating the tumor microenvironment has also been associated with clinical benefit from ICIs. Tumeh and colleagues (29) analyzed the relationship between TILs and response to pembrolizumab in patients with melanoma; the results showed higher CD8^+^ T cell densities at the invasive margin and within the tumor parenchyma in responding patients than in patients with disease progression. TILs and components associated with TILs as prognostic biomarkers and their potential value in predicting response to ICIs in sarcoma have been explored (30). Therefore, other factors such as TIME should also be considered when evaluating response to ICIs in NSCLC patients with different EGFR mutation subtypes. Although some of theoretical conditions suitable for ICIs therapy were showed in several of EGFR mutation subtypes in this research, however, no clinical cohort of ICIs treatment was established in this manuscript. Obviously, further studies need to be carried out for clinical verification.

In addition to genetic and immunologic differences, differences in prognosis should be considered for each subtype of EGFR mutation. The prognostic significance of TILs has been confirmed in several solid tumors, but less is known about whether components of the TIME have prognostic value in NSCLC, especially in each EGFR-mutated subtype. Only 98 patients were available for follow-up, and among them, data for analysis of TIME in each EGFR mutation subtype were not available. Therefore, we only analyzed the TIME as a prognostic biomarker in patients without and with EGFR mutation (EGFR-/+) and found different prognostic biomarkers (**Figure 6**). Hence, based on the above results of genetic and immunologic differences, we infer that the difference in prognostic biomarkers is not limited to EGFR mutation. In general, each subtype may have different prognostic factors, but a larger sample may be needed for confirmation.

In conclusion, EGFR positive NSCLC is one kind of complex disease, this research demonstrated genetic and immunological heterogeneity, and the differences in prognosis. These results suggested that NSCLC patients with various EGFR mutation should be treated and managed differently in clinical practice. ICIs may not be excluded for total EGFR positive NSCLC. Of course, more studies are needed, especially interventional studies are needed for further confirmation.

## Data Availability Statement

The datasets presented in this article are not readily available because sequencing data contains sequencing algorithm and other core trade information of 3D Medicines Inc. Requests to access the datasets should be directed to the corresponding author Hushan Zhang (E-mail address: 15111010041@fudan.edu.cn/hush111@126.com).

## Ethics Statement

The studies involving human participants were reviewed and approved by Ethics Committee of the First Affiliated Hospital of Kunming Medical University, Ethics Committee of the First People’s Hospital of Anning, Yunnan province. The patients/participants provided their written informed consent to participate in this study.

## Author Contributions

HZ, YML, KW, YQL, and XW put forward the content of the paper. HZ wrote the manuscript. The others literature and clinical data were collected and reviewed. All authors read and approved the final manuscript.

## Funding

This research was supported by the following funding: Scientific Research Foundation of Education Department of Yunnan Province, item number: 2021J0361 and 2021J0362.

## Conflict of Interest

Author DH, YB, HZ was employed by 3D Medicines Inc.

The remaining authors declare that the research was conducted in the absence of any commercial or financial relationships that could be construed as a potential conflict of interest.

## Publisher’s Note

All claims expressed in this article are solely those of the authors and do not necessarily represent those of their affiliated organizations, or those of the publisher, the editors and the reviewers. Any product that may be evaluated in this article, or claim that may be made by its manufacturer, is not guaranteed or endorsed by the publisher.

## Supplementary Material

Supplementary Figure 1 Proportion of MSI-H in NSCLC patients with and without EGFR mutation.

## Abbreviations

CAP, College of American Pathologists; CLIA, Clinical Laboratory Improvement Amendment; EGFR, Epidermal growth factor receptor; FFPE, Formalin-fixed paraffin-embedded; ICI, Immune checkpoint inhibitors; IHC, Immunocytochemistry; NGS, Next generation sequencing; NSCLC, Non-small cell lung cancer; PFS, Progression-free survival; PD-1(L1), Programmed cell death protein 1/programmed cell death ligand 1; TILs, Tumor-infiltrating lymphocytes; TIME, Tumor immune microenvironment; TKI, Tyrosine kinase inhibitor; TMB, Tumor mutation burden; WES, Whole exome sequencing; 19del, EGFR exon 19 deletion.
