# Supplementary figures and images for: Various Subtypes of EGFR Mutations in Patients With NSCLC Define Genetic, Immunologic Diversity and Possess Different Prognostic Biomarkers
Source: Front Immunol. 2022 Feb 21;13:811601. doi: 10.3389/fimmu.2022.811601 (PMC8899028; doi:10.3389/fimmu.2022.811601)

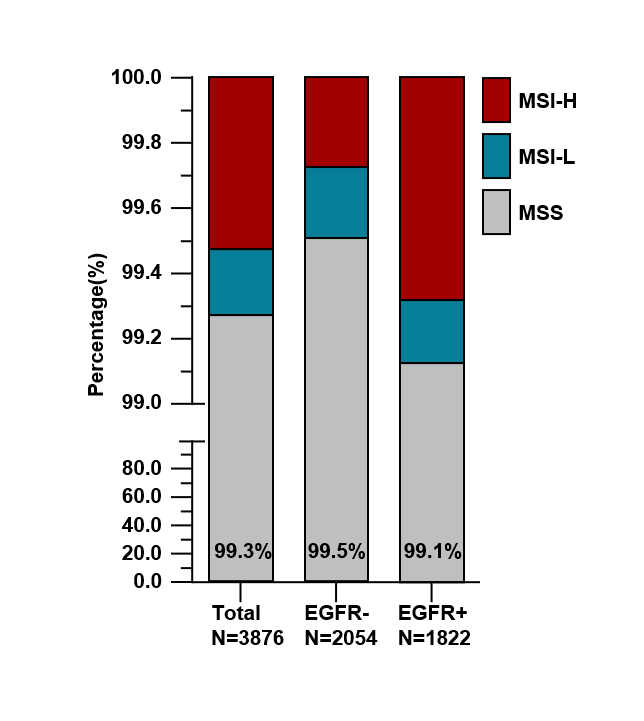

Supplement: Supplementary Figure 1 — Proportion of MSI-H in NSCLC patients with and without EGFR mutation. [file Image_1.tif]
