# Supplementary material for: Various Subtypes of EGFR Mutations in Patients With NSCLC Define Genetic, Immunologic Diversity and Possess Different Prognostic Biomarkers
Source: Front Immunol. 2022 Feb 21;13:811601. doi: 10.3389/fimmu.2022.811601 (PMC8899028; doi:10.3389/fimmu.2022.811601)
Supplement: Supplementary file 2 [file Table_1.docx]

**Table S1. List of genes of the 733-gene panel**

| ABL1 | CDX2 | FGFR4 | MLH1 | PTEN | VEGFA | JMJD1C | TRIM37 | BCL11A | EZR | TBL1XR1 | PLXNB1 | LIG1 | RNF168 | POLD3 |
| --- | --- | --- | --- | --- | --- | --- | --- | --- | --- | --- | --- | --- | --- | --- |
| ACVR2A | CHD2 | FH | MLLT3 | PTK6 | VHL | LMO1 | TSHR | BCL11B | FAT4 | TCF7L2 | SPRED1 | LIG3 | RNF4 | POLD4 |
| AFF3 | CHEK1 | FHIT | MPL | PTPRD | NSD3 | LZTR1 | UROD | BCORL1 | FUBP1 | TCL1A | ERF | LIG4 | RNF8 | POLE2 |
| AKT1 | CHEK2 | FLCN | MRE11A | RAC1 | ZNF479 | MAX | WAS | BIRC3 | FUS | TET1 | RPS6KA3 | MAD2L2 | RPA1 | POLE4 |
| AKT2 | CHIC2 | FLT1 | MSH2 | RAD50 | ZNRF3 | MEN1 | WRN | BRD4 | GAS7 | TFE3 | GSK3B | MBD4 | RPA2 | PPP4R1 |
| AKT3 | CIC | FLT3 | MSH3 | RAD51 | ABCB11 | MTAP | WT1 | CACNA1D | H3F3A | TNFAIP3 | NOTCH3 | MDC1 | RPA3 | PPP4R3A |
| ALK | CIITA | FLT4 | MSH6 | RAD51C | APOBEC3B | MUTYH | XPA | CALR | HIF1A | USP8 | NOTCH4 | MGMT | RPA4 | PPP4R3B |
| ANK1 | CRBN | FOXA1 | MTOR | RAF1 | AXIN2 | NBN | XPC | CAMTA1 | HIP1 | WIF1 | ALKBH2 | MLH3 | RRM2B | PPP4R4 |
| APC | CRLF2 | FRS2 | MYC | RARA | BARD1 | NHP2 | XRCC2 | CANT1 | HNRNPA2B1 | XPO1 | ALKBH3 | MMS19 | SETMAR | RAD9B |
| AR | CRNKL1 | G6PD | MYCN | RB1 | BMPR1A | NME1 | HOXB13 | CARD11 | HOXA11 | ZFHX3 | APEX1 | MNAT1 | SEM1 | RBX1 |
| ARAF | CRTC3 | GATA3 | MYD88 | RET | BUB1B | NOP10 | BCL2L1 | KNL1 | IL6ST | ACVR1B | APEX2 | MPG | SHPRH | RFC1 |
| AREG | CSF1R | GLI2 | NF1 | RGS7 | CDC73 | NTHL1 | BCL6 | CASP8 | KDM6A | ARID1B | CENPS | MSH4 | SMUG1 | RFC2 |
| ARHGAP5 | CSF3R | GNA11 | NF2 | RICTOR | CDKN1C | PHOX2B | CDK8 | CBFA2T3 | KEAP1 | DNMT1 | APLF | MUS81 | SPO11 | RFC3 |
| ARID1A | CTNNB1 | GNAQ | NFE2L2 | RNF43 | CEBPA | PMS1 | FOXP1 | CBFB | KLF4 | FOXL2 | APTX | NEIL1 | TDG | RFC4 |
| ARNT | CTNND2 | GNAS | NFIB | ROS1 | COL7A1 | POLH | GRIN2A | CBLB | LCK | GATA1 | ATRIP | NEIL2 | TDP1 | TELO2 |
| ASXL1 | CUL3 | HDAC2 | NKX2-1 | RPTOR | CTR9 | POLQ | IKBKE | CCDC6 | LEF1 | HIST1H3B | FAAP100 | NEIL3 | TDP2 | TIMELESS |
| ATM | CYSLTR2 | HEY1 | NOTCH1 | RUNX1 | CXCR4 | POT1 | MEF2B | CCNB1IP1 | LIFR | KDM5C | FAAP24 | NHEJ1 | TOP3A | TMEM189 |
| ATR | DDR2 | HGF | NOTCH2 | SDC4 | CYLD | PRDM9 | NFKBIA | CD79A | MAPK1 | MAP3K1 | FAAP20 | NUDT1 | TOP3B | WDR48 |
| AURKA | DICER1 | HOOK3 | NPM1 | SDHC | DDB2 | PRF1 | PIK3CD | CD79B | MED12 | KMT2C | MPLKIP | NABP2 | TOPBP1 | GFI1 |
| AXL | DNMT3A | HRAS | NRAS | SERPINB3 | DIS3L2 | PRKAR1A | SRC | CDH11 | NAB2 | NCOR1 | CCNH | OGG1 | TP53BP1 | CYP17A1 |
| B2M | DPYD | IDH1 | NRG1 | SETD2 | DKC1 | PRSS1 | BTG1 | CHD4 | NCOR2 | PHF6 | CDK7 | PARP1 | TREX1 | ELF3 |
| BAP1 | EGFR | IDH2 | NTRK1 | SF3B1 | DOCK8 | PTPN11 | DIS3 | CLIP1 | NDRG1 | PPP2R1A | CETN2 | PARP2 | TREX2 | SGK1 |
| BAZ1A | EPHA2 | IGF1R | NTRK2 | SH2B3 | DROSHA | PTPN13 | EED | CLTCL1 | NONO | PRDM1 | CHAF1A | PARP3 | UBE2A | GSTT1 |
| BCL2 | EPHA3 | IGF2 | NTRK3 | SLC29A1 | ELANE | RAD51B | GNA13 | CNBP | PAX3 | SOCS1 | CLK2 | PCNA | UBE2B | AEN |
| BCOR | ERBB2 | IL7R | PAK1 | SMAD4 | EPCAM | RAD51D | NT5C2 | CNOT3 | PAX7 | SOX9 | DCLRE1A | PNKP | UBE2N | CCNO |
| BLM | ERBB3 | INPP4B | PALB2 | SMARCA1 | ERCC3 | RECQL | PPP2R2A | CREB3L1 | PAX8 | TRAF7 | DCLRE1B | POLB | UBE2T | CENPX |
| BMP5 | ERBB4 | ITGAV | PAX5 | SMARCA4 | ERCC5 | RECQL4 | NSD2 | CREB3L2 | PER1 | IKZF1 | DCLRE1C | POLI | UBE2V2 | CUL4A |
| BRAF | ERCC1 | JAK1 | PBRM1 | SMARCB1 | ETV6 | RFWD3 | EPHA7 | CREBBP | PICALM | MYCL | DDB1 | POLK | UNG | CUL5 |
| BRCA1 | ERCC2 | JAK2 | PDCD1LG2 | SMO | EXT1 | RHBDF2 | GLI1 | CRTC1 | PIM1 | NCOA3 | DMC1 | POLL | USP1 | DNTT |
| BRCA2 | ERCC4 | JAK3 | PDGFB | SRGAP3 | EXT2 | SBDS | MYB | CTCF | POU2AF1 | CDK2 | DUT | POLM | XAB2 | ELOA |
| BRIP1 | ERCC6 | JUN | PDGFRA | SRSF2 | FAH | SDHA | NRG3 | CUX1 | POU5F1 | LATS1 | EME1 | POLN | XRCC1 | HUS1B |
| BTK | EREG | KCNJ5 | PDGFRB | STAG2 | FANCD2 | SDHAF2 | NUP93 | DAXX | PPP6C | LATS2 | EME2 | PRKDC | XRCC3 | PER2 |
| CARS | ESR1 | KDR | PDPK1 | STK11 | FANCE | SDHB | PTK2 | DDIT3 | PRDM16 | YAP1 | ENDOV | PRPF19 | XRCC4 | PER3 |
| CBL | EWSR1 | KIT | PIK3CA | SUZ12 | FANCF | SDHD | RXRA | DDX10 | PREX2 | TEAD2 | ERCC8 | RAD1 | XRCC5 | MSH5 |
| CCND1 | EZH2 | KMT2A | PIK3CB | SYK | FANCI | SERPINA1 | SMARCA2 | DDX3X | PRKACA | MGA | EXO1 | RAD18 | XRCC6 | PARP4 |
| CCND2 | FAM135B | KMT2D | PIK3R1 | TBX3 | FANCL | SETBP1 | TYK2 | DDX5 | PTPRT | HES1 | FAN1 | RAD23A | ABRAXAS1 | POLE3 |
| CCND3 | FAM47C | KRAS | PIK3R2 | TCF3 | FANCM | SH2D1A | ZNF750 | DDX6 | QKI | KDM5A | FANCB | RAD23B | FRK | PPP4R2 |
| CCNE1 | FANCA | LASP1 | PLCG2 | TERT | FAS | SHOC2 | ABI1 | DNM2 | RAD21 | SPEN | GEN1 | RAD52 | BIRC5 | SLX1A |
| CD274 | FANCC | LMNA | PML | TET2 | FEN1 | SLC25A13 | ACKR3 | EBF1 | RANBP2 | THBS2 | GTF2H1 | RAD54B | EMSY | RAD54L2 |
| CDH1 | FANCG | LRP1B | PMS2 | TMEM127 | GALNT12 | SLX4 | ACSL3 | EIF3E | RAP1GDS1 | CUL1 | GTF2H3 | RAD54L | CRKL | RFC5 |
| CDH10 | FAT1 | MAP2K1 | POLD1 | TMPRSS2 | GATA2 | SOS1 | ACVR1 | EIF4A2 | RBM10 | HDAC1 | GTF2H4 | RAD9A | EPHB1 | HMGA2 |
| CDK12 | FBXW7 | MAP2K2 | POLE | TOP2A | GBA | SPOP | AFF4 | ELF4 | RHOA | MLST8 | GTF2H5 | RBBP8 | GLI3 | TSPAN31 |
| CDK4 | FES | MAP2K4 | POLG | TP53 | GJB2 | SPRTN | AMER1 | ELK4 | RHOH | PIK3R3 | H2AFX | RDM1 | IRS2 | MYOD1 |
| CDK6 | FGF19 | MCL1 | PPARG | TPMT | GPC3 | SRY | ARID2 | ELL | RNF213 | RHEB | HELQ | RECQL5 | RUNX1T1 | CHD1 |
| CDKN1A | FGF3 | MDM2 | PPM1D | TSC1 | GREM1 | STAT3 | ATP1A1 | EP300 | SFPQ | RPS6KB1 | HFM1 | REV1 | SLIT2 | ZBTB16 |
| CDKN1B | FGF4 | MDM4 | PRCC | TSC2 | HFE | SUFU | ATP2B3 | EPAS1 | SLC34A2 | GRB2 | HLTF | REV3L | SOX2 | PCDH9 |
| CDKN2A | FGFR1 | MECOM | PRKCH | U2AF1 | HMBS | TGFBR1 | ATRX | EPS15 | SLC45A3 | RIT1 | HMGB1 | RIF1 | SPTA1 | PLXNA1 |
| CDKN2B | FGFR2 | MET | PSIP1 | UGT1A1 | HNF1A | TGFBR2 | AXIN1 | ERC1 | SMAD2 | RASA1 | HUS1 | RMI1 | ZNF217 |  |
| CDKN2C | FGFR3 | MITF | PTCH1 | USP6 | ITK | TP63 | BCL10 | ETNK1 | SMAD3 | ERRFI1 | UVSSA | RMI2 | ZNF703 |  |
